# Identification and functional characterization of novel transcriptional enhancers involved in regulating human *GLI3* expression during early development

**DOI:** 10.1111/dgd.12239

**Published:** 2015-10-14

**Authors:** Saneela Anwar, Rashid Minhas, Shahid Ali, Nicholas Lambert, Yasuhiko Kawakami, Greg Elgar, Syed Sikandar Azam, Amir Ali Abbasi

**Affiliations:** ^1^ National Center for BioinformaticsComputational Biology LabFaculty of Biological SciencesQuaid‐i‐Azam UniversityIslamabad45320Pakistan; ^2^ National Center for BioinformaticsProgram of Comparative and Evolutionary GenomicsFaculty of Biological SciencesQuaid‐i‐Azam UniversityIslamabad45320Pakistan; ^3^ Department of GeneticsCell Biology and DevelopmentUniversity of MinnesotaMinneapolisMinnesota55455USA; ^4^ The Francis Crick InstituteMill Hill LaboratoryThe RidgewayLondonNW7 1AAUK

**Keywords:** *Cis*‐regulators, conserved non‐coding elements, enhancer, fin, *GLI3*

## Abstract

The zinc‐finger transcription factor GLI3 acts as a primary transducer of Sonic hedgehog (Shh) signaling in a context‐dependent combinatorial fashion. *GLI3* participates in the patterning and growth of many organs, including the central nervous system (CNS) and limbs. Previously, we reported a subset of human intronic *ci*s‐regulators controlling many known aspects of endogenous *Gli3* expression in mouse and zebrafish. Here we demonstrate in a transgenic zebrafish assay the potential of two novel tetrapod‐teleost conserved non‐coding elements (CNEs) docking within *GLI3* intronic intervals (intron 3 and 4) to induce reporter gene expression at known sites of endogenous *Gli3* transcription in embryonic domains such as the central nervous system (CNS) and limbs. Interestingly, the cell culture based assays reveal harmony with the context dependent dual nature of intra‐*GLI3* conserved elements. Furthermore, a transgenic zebrafish assay of previously reported limb‐specific *GLI3* transcriptional enhancers (previously tested in mice and chicken limb buds) induced reporter gene expression in zebrafish blood precursor cells and notochord instead of fin. These results demonstrate that the appendage‐specific activity of a subset of *GLI3*‐associated enhancers might be a tetrapod innovation. Taken together with our recent data, these results suggest that during the course of vertebrate evolution *Gli3* expression control acquired a complex *cis*‐regulatory landscape for spatiotemporal patterning of CNS and limbs. Comparative data from fish and mice suggest that the functional aspects of a subset of these *cis*‐regulators have diverged significantly between these two lineages.

## Introduction

The zinc finger transcription factor *GLI3* (a member of the GLI family) acts as an antagonist or mediator for the Sonic hedgehog (Shh) signaling cascade in a context‐dependent manner during vertebrate embryogenesis (Ruppert *et al*. 1998; Coy *et al*. 2011). GLI3 is an important developmental regulator and is dynamically expressed in the brain, axial, appendicular, and craniofacial structures, as well as within various visceral organs prenatally, postnatally, and in adult life (Mo *et al*. 1997; Motoyama *et al*. 1998; McDermott *et al*. 2005; Lebel *et al*. 2007). Thus, the spatio‐temporal expression of Gli3 is highly orchestrated and regulated, and its aberrant expression can lead to several developmental defects (Hui & Angers 2011). A multitude of studies in mice and other model organisms have proven that a GLI‐code, the interplay of GLI proteins (GLI1, GLI2 and GLI3), and their temporally fine‐tuned expression in adjacent domains together provide a basic tool that is used at various stages of embryonic development (Ruiz I Altaba *et al*. 2007).

Congenital abnormalities associated with human *GLI3* are listed under the term “GLI3 morphopathies”, including Greig cephalopolysyndactyly syndrome (GCPS), non‐syndromic polydactyly, Pallister Hall syndrome (PHS), acrocallosal syndrome, preaxial polydactyly type IV (PPD‐IV) and postaxial polydactyly type A (PAPA) (Vortkamp *et al*. 1991; Kang *et al*. 1997; Radhakrishna *et al*. 1997, 1999; Elson *et al*. 2002). Moreover, *GLI3* is also associated with oral‐facial‐digital syndrome (OFDS) and Opitz syndrome (OS) (Liu *et al*. 2001; Johnston *et al*. 2010). A dominant developmental syndrome, GCPS with polydactyly and craniofacial abnormalities, is linked with large deletions, translocations and truncating mutations resulting in functional haploinsufficiency of *GLI3* (Shin *et al*. 1999; Johnston *et al*. 2010). Mutations affecting murine *Gli3*, such as an extra toe (*Xt*), anterior digit deformity, and polydactyly Nagoya (*Pdn*), serve as models for GLI3 morphophathies (Pohl *et al*. 1990; Schimmang *et al*. 1992, 1994; Hui & Joyner 1993). The limbs of *Gli3* ^−^/^−^ mutant mouse embryos show severe polydactyly characterized by many un‐patterned digits, a loss of A‐P polarity, and the absence of apoptosis in inter‐digital regions (Welscher *et al*. 2002b).

The pleiotropy of *GLI3* is indicative of a highly complex and sophisticated *cis*‐acting regulatory network governing GLI3 expression in the correct spatiotemporal manner during embryonic development and post‐natally. Previously, a set of 12 human‐fugu ancient gene regulatory elements were identified that participate in the spatio‐temporal expression of *GLI3* (Abbasi *et al*. 2007, 2010, 2013). Eleven of these act as enhancers or repressors in a cell‐type dependent manner in cultured cells (Abbasi *et al*. 2007; Paparidis *et al*. 2007). *GLI3*‐CNEs, having regulatory potential in human cell lines, were also tested in zebrafish and mouse embryos (Abbasi *et al*. 2010). Reporter gene expression in transgenic animal models is observed in a multitude of organs, including brain, spinal cord, limbs, eye, craniofacial structures, and internal organs (Abbasi *et al*. 2007, 2010, 2013).

In the present study, we identify two novel tetrapod‐teleost conserved intronic regions at the *GLI3* locus using comparative genomics. Our results obtained through *Tol2*‐based transgenesis in zebrafish demonstrate that these novel intronic CNEs act as tissue‐specific enhancers and regulate reporter gene expression in zebrafish hindbrain and pectoral fin. Interestingly, *in vitro* studies (luciferase reporter assays) of these novel regulators reveal the distinct activities of intra‐*GLI3* conserved genomic intervals. Furthermore, we test the regulatory potential of previously reported *GLI3* associated limb specific *cis*‐regulators, CNE6 and CNE11, in zebrafish (Abbasi *et al*. 2010). Taken together our studies, based on comparative functional data from fish and mice, suggest that appendage‐specific activity of a subset of *GLI3*‐associated *cis*‐regulators might be a tetrapod innovation.

## Materials and methods

### Identification of conserved non‐coding elements at the *GLI3* locus

The human *GLI3* genomic sequence was obtained from the ENSEMBL genome browser along with orthologous sequences from mouse (NCBIM37), chicken (Galgal4), lizard (AnoCar2.0), fugu (Fugu4) and zebrafish (Zv9). Multi‐species sequence comparisons were performed using the Shuffle LAGAN (SLAGAN) alignment tool kit (Brudno *et al*. 2003). The human sequence was used as a baseline and annotated by exon/intron information available at ENSEMBL genome browser. The SLAGAN alignment was visualized using VISTA (Mayor *et al*. 2000). The conservation was measured using a 50 bp window and a cutoff score of 50% identity.

### *In silico* mapping of conserved transcription factor binding sites (TFBSs)

To identify conserved TFBSs for each CNE, the orthologous sequences of terrestrial and non‐terrestrial vertebrates were retrieved from the Ensembl genome database. Each of the CNEs with orthologous sequences were analyzed using the MEME motif discovery algorithm (Bailey *et al*. 2009). MEME is a position weight matrixes (PWM) based algorithm that identifies over‐represented motifs in the query data et. The criteria for minimum length was set from 6 to 12 bp. The identified motifs of each CNE were further characterized using the STAMP tool (Mahony & Benos 2007) to determine known transcription factors against TRANSFAC (v11.3) library (Matys *et al*. 2003). Each of the specified transcription factors were then chosen for endogenous gene expression (RNA *in‐situ* hybridization) studies using the Mouse Genome Informatics database (http://www.informatics.jax.org/).

### Luciferase reporter assay

For the luciferase reporter assay, CNEs were cloned into pGL3 with minimum TK promoter, and confirmed by nucleotide sequencing. The NIH3T3 cell line is maintained in Dulbecco's modified eagle medium (DMEM) supplemented with 10% fetal bovine serum (FBS) and 10 units/mL penicillin/streptomycin. Cells were plated at 3 × 10^4^/well into a 48 well plate, and luciferase reporter constructs (100 ng) and pRL‐TK (5 ng) were transfected using Fugene 6 (Promega), according to the manufacturer's instructions. Cells were harvested 40–44 h after transfection and luciferase activities measured using the Dual Luciferase Reporter Assay System (Promega), according to the manufacturer's instructions. Experiments were done in triplicate, and the results represented shown as average standard +/− deviation. Statistical significance was evaluated by *t*‐test.

### Zebrafish transgenic assays

We used two approaches, a co‐injection assay and a *Tol2* transposon‐based assay to test activities of CNEs *in vivo* using zebrafish embryos. Zebrafish were bred and raised according to standard protocols (Kimmel *et al*. 1995). The co‐injection assays were performed, as described previously (Woolfe *et al*. 2005; Minhas *et al*. 2015). For the preparation of DNA and micro‐injection, CNEs were polymerase chain reaction (PCR)‐amplified from human genomic DNA. The reporter expression cassette consisting of EGFP under the control of a minimal promoter from the mouse *β*‐globin gene was amplified from plasmid vector by PCR (Thermo Scientific DNA Taq), and purified using the PureLink PCR purification kit, according to manufacturer's instructions (Life Technologies). PCR purified product of CNEs (30 ng/μL) and *β*‐globin‐GFP promoter‐reporter cassette (15 ng/μL) were combined, and 0.5% phenol red (Sigma) was used as a tracer dye. The injected embryos were raised at 28.5°C in 1× embryo medium containing 0.003% PTU to prevent pigmentation. The zebrafish embryos were dechorionated manually by fine forceps at day 2 and anaesthetized by Tricaine.

The *Tol2* system is based on a transposon system, which allows for efficient transgene integration (Kawakami 2007). To test CNEs using a *Tol2* GFP system (Fisher *et al*. 2006), the CNEs were first amplified with a final 10–30 min extension step. The freshly amplified PCR (~500 ng/μL) products were cloned into pCR8/GW/TOPO vector (Life Technologies) to make entry clones (Pauls *et al*. 2012; Chen *et al*. 2014). Orientation screening to determine the sense strand was followed by a LR (attL and attR) recombination reaction between Topo entry clone (~100 ng/μL) and destination vector pGW_cfosEGFP (~100 ng/μL); Gateway LR Clonase II enzyme (Life Technologies) was used. The destination clones consisting of CNEs and a minimal *c*‐*fos* promoter were sequenced for confirmation of a positive orientation into the transposon construct. The purified transposon construct (25 ng/μL), 0.5 μL transposase RNA (175 ng/μL), and 0.5 μL phenol red stock, were injected into one‐cell stage zebrafish embryos.

### Images and screening

The transgenic embryos were screened after approximately 24 and 48 hpf for GFP signals using an Olympus IX71 inverted fluorescence microscope. Photographs were taken with an Olympus DP72 camera.

## Results

### Identification of CNEs at the *GLI3* locus by comparative sequence analysis

**Figure 1:**
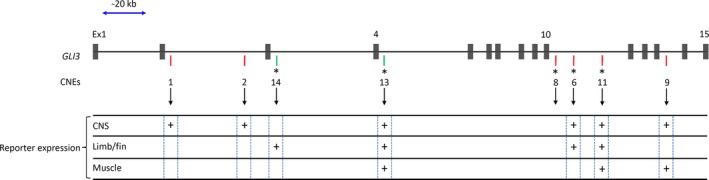
Distribution of conserved non‐coding elements (CNEs) in the human *GLI3* locus. Schematic representation of human *GLI3* locus with coding regions in black rectangles and CNEs in green and red lines. CNEs presented with green lines are newly identified, whereas those shown in red lines were previously identified (Abbasi *et al*. 2007, 2010; Paparidis *et al*. 2007). The subset of five intronic CNEs selected for functional analysis in this report are presented with a “*” mark. CNEs that drove the reporter gene expression in various domains (CNS, limb/fin and muscle) are marked as “+”. Exons and CNEs are drawn according to the approximate scale. A blue arrow on the top depicts the approximate scale. Ex, exon; kb, kilobase.

Previously, we identified 12 anciently conserved genomic elements in the intronic intervals of human *GLI3* through human‐fish comparative sequence analysis (Abbasi *et al*. 2007). *Cis*‐regulatory potential of 11 of these *GLI3* intronic regions was elucidated by using *in vitro* and *in vivo* assays (Fig. S1) (Abbasi *et al*. 2007, 2010, 2013; Paparidis *et al*. 2007). In the present study, we extended our previous work and analyzed the sequence alignments more extensively to capture any remaining anciently conserved *GLI3* intronic intervals (Fig. S1). This careful comparative sequence analysis pinpointed two novel conserved sequences within intronic intervals of human *GLI3,* thereby named CNE13 (intron‐4) and CNE14 (intron‐3). Taken together with our previous investigations, human *GLI3* intronic intervals thus harbor 14 ancient conserved CNEs in total with at least 50% identity over a 50 bp window (Fig. 1 and Fig. S1).

### Functional assays of *GLI3*‐associated CNEs in transgenic zebrafish embryos

The regulatory potential of a selected subset of novel CNEs identified in the present study (CNE8, 13 and 14) was tested independently in zebrafish embryos by usiung two independent strategies: exploiting a co‐injection assay (*β*‐globin promoter) and directly cloning into a *Tol2* vector (*c‐fos* promoter) (Woolfe *et al*. 2005; Fisher *et al*. 2006). In contrast, previously identified limb‐specific CNE6 and 11 (which have shown transcriptional activity in developing limb bud of mice and chicken) (Abbasi *et al*. 2010) were only tested using a *Tol2* vector with a *c‐fos* minimal promoter in zebrafish transgenic assays. The details of CNEs selected for functional analyses are depicted in Figure 1 and also listed in Table 1. The results from both assays are highly reproducible, while GFP expression is generally stronger using the *Tol2* strategy due to efficient integration and reduced mosaicism (Fisher *et al*. 2006; Kawakami 2007).

**Table 1:** Conserved non‐coding elements (CNEs) from introns of human *GLI3* selected for functional analysis

Element	Region	Amplicon coordinates Chr7	Amplicon size	Conservation depth 50% >50 bp	Co‐injection assay	*Tol2* assay	Expression in fin with *Tol2*	Expression in mice limb	Conserved putative TFBS
CNE 6	Intron10	42051639–42052500	862 bp	179 bp (Fugu)	+ve	+ve	−ve	+ve	OCT1, PPARA, TBXS, PAX4
CNE8	Intron 10	42059671–42060244	574 bp	144 bp (Fugu)	−ve	−ve	−ve	†	†
CNE11	Intron 10	42035686–42036870	1185 bp	129 bp (Fugu)	+ve	+ve	−ve	+ve	SMAD3, LEF1B
CNE13	Intron 4	42115076–42115699	624 bp	88 bp (Fugu)	+ve	+ve	+ve	†	NKX2‐2, FOXP3,, TTK, SPI.1, HNF4, HSF2, LMAF, DMRT2, DMRT4, AIRE, ARID5B, SOX17, NKX2‐5, EGR1, AR
CNE14	Intron 3	42185602–42187508	1907 bp	357 bp (Lizard)	−ve	+ve	+ve	†	GATA1, REST, CREB1, HNF4A, NR2F1, PPARA, YY1, CDC5, HSF, ANT, GCNF PAX6, HSF1, NFKB1

^†^Not analyzed yet. Location, size, coordinates and putative conserved transcription factor binding sites are indicated. +ve indicates the elements that induced green fluorescent protein (GFP) expression in zebrafish (co‐injection, *Tol2*) and mice (*LacZ*) embryos, whereas –ve indicates those that do not drive significant GFP expression.

### CNE13 drives expression predominantly in the hindbrain, notochord and pectoral fin bud

**Figure 2:**
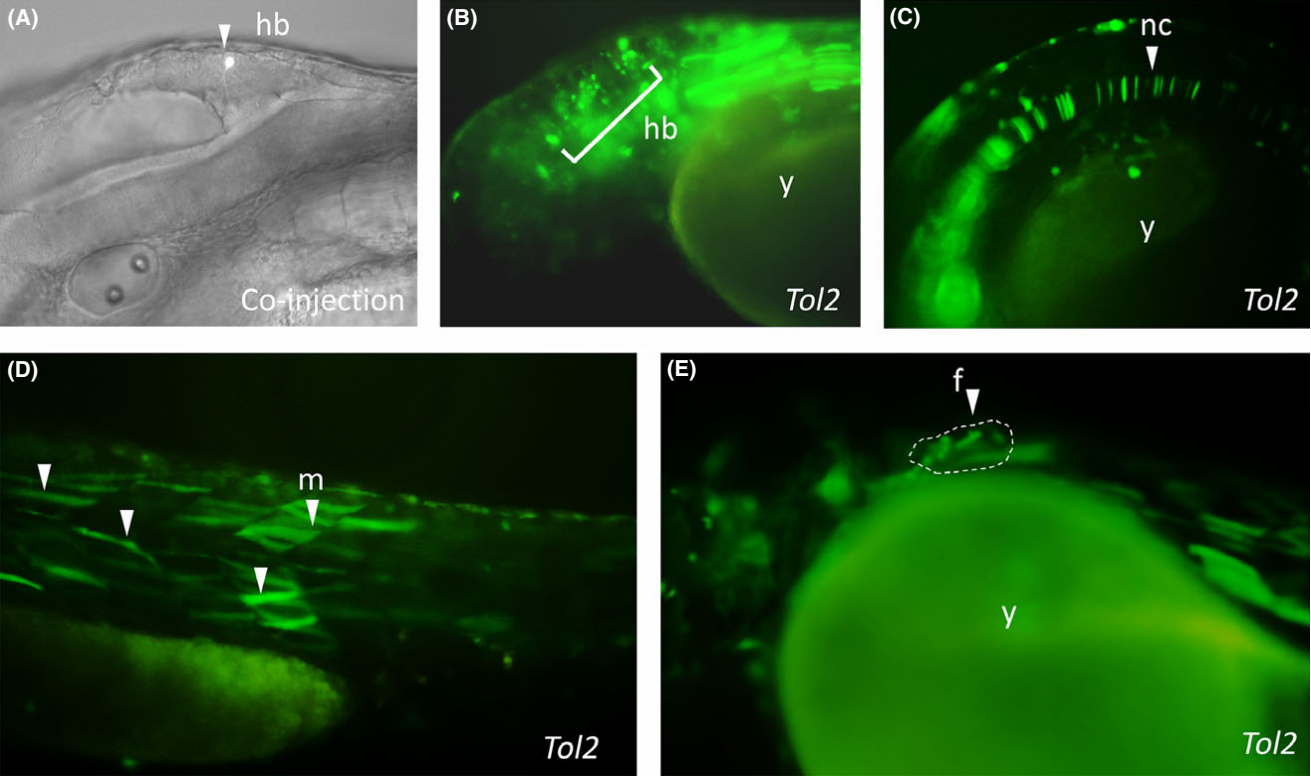
Green fluorescent protein (GFP) expression induced by conserved non‐coding element 13 (CNE13) in zebrafish embryos at day 2 and day 3 of development. CNE13 drives GFP expression pattern in live embryos at day 2 and day 3. Embryo C is approximately 26–33 hpf, while embryos A, B, D and E are approximately 48–56 hpf. Orientation of the embryos is anterior to left, dorsal to top, with lateral views. Arrowheads and marked area indicate GFP expressing cells in hindbrain at day 3 by co‐injection (A) and *Tol2* (B), as well as in the developing notochord (C), muscle fibers (D) and pectoral fin (E). hpf, hours postfertilization; hb, hindbrain; nc, notochord; m, muscle; y, yolk.

This novel element (CNE13) is deeply conserved (human‐fish) and resides within intron‐4 of human *GLI3*. The human genomic segment of 624 bp spanning the human‐fish conserved core sequence of 88 bp was co‐injected with a GFP reporter into zebrafish and then monitored for enhancer activity at set time points. From this assay it appears that CNE13 directs reporter gene expression prominently in the hindbrain after approximately 48–56 hpf (Fig. 2A and Fig. S2). To further confirm results obtained with the co‐injection experiment, the *Tol2* based assay was also used on CNE13. In the *Tol2* assay, the major activity domain for CNE13 was again hindbrain territory (Fig. 2B). Reproducible GFP expression in the hindbrain with two independent assays using independent promoter systems strongly suggests that CNE13 plays a role in neuronal development. In the *Tol2* assay, notable reporter gene expression was also detected in the notochord, muscle cells and pectoral fin (Fig. 2C–E). The CNE13 induced GFP signal in the pectoral fin, which appeared after 48 hpf, was robust and reproducible. It is notable that with the co‐injection assay, CNE13 was unable to upregulate GFP expression in the developing pectoral fin, possibly due to high levels of mosaicism associated with this strategy (Woolfe *et al*. 2005; Abbasi *et al*. 2007; Kawakami 2007; Minhas *et al*. 2015).

### CNE14 drives GFP expression exclusively in the pectoral fin

**Figure 3:**
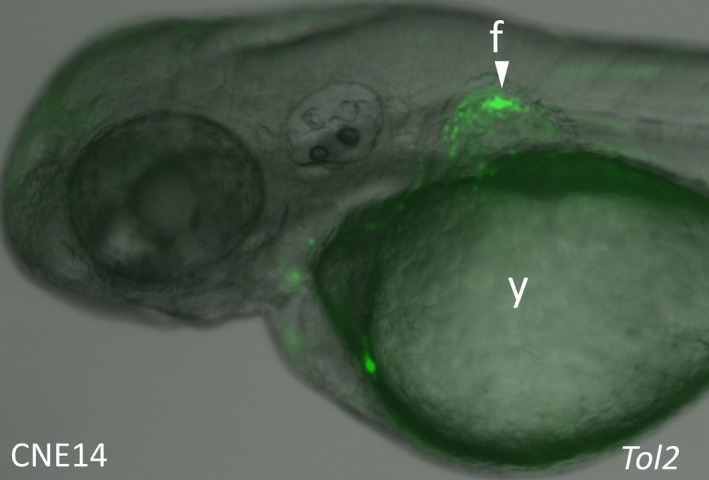
Conserved non‐coding element 14 (CNE14) is a fin‐specific enhancer. An Image of a live zebrafish embryo at day 3, lateral views, anterior to left, dorsal to top. Arrowhead indicates green fluorescent protein (GFP) expressing cells. CNE14 induced GFP expression exclusively in pectoral fin. f, fin; y, yolk.

The second novel evolutionarily conserved intronic interval identified through comparative sequence analysis in the present study was named CNE14. This element resides within intron‐3 of *GLI3* and is highly conserved among diverse lineages of tetrapods. When tested in a co‐injection experiment, a genomic segment of approximately 2.0 Kilobase pair (kbp) was unable to induce reporter gene expression in fish embroys at day 2 or day 3 of development. However, when tested using the *Tol2* strategy, CNE14 was able to drive robust and reproducible GFP expression in the pectoral fin after 48 hpf (Fig. 3). Interestingly, CNE14 induced reporter expression was limited to the pectoral fin only, whereas no significant expression was observed in any of the other developmental domains of zebrafish embryos at day 2 or day 3 of development. Failure to detect reporter expression with co‐injection assay again illuminates the fact that this strategy is not suitable for detecting the activity of limb‐specific regulators.

### CNE6 and CNE11 limb‐specific enhancers did not induce reporter gene expression in the zebrafish fin

Previously, we have shown in chicken and mouse transgenic embryos that the human‐fish evolutionarily conserved *GLI3*‐intronic enhancers named CNE6 and CNE11 autonomously control individual aspects of GLI3 expression in the developing limb skeletal structures. For instance, the prominent activity domain of CNE6 was autopod‐specific, whereas CNE11 appeared to be a stylopod/zeugopod specific *cis*‐regulator (Abbasi *et al*. 2010).

**Figure 4:**
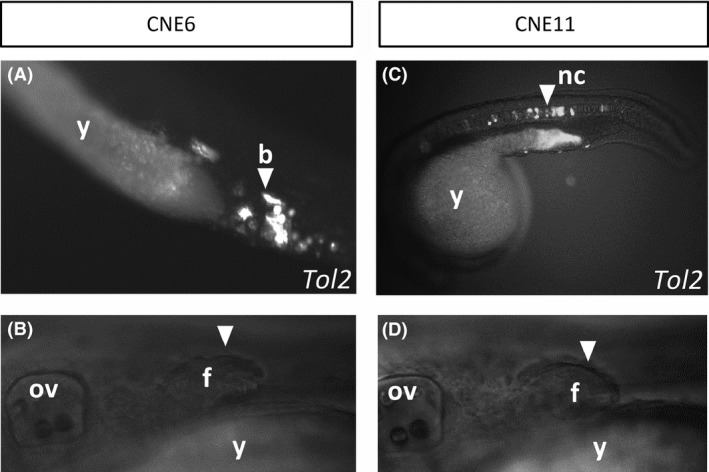
Limb‐specific enhancers cannot upregulate reporter gene expression in the pectoral fin with Tol2 assay. Images of live zebrafish embryos at day 2, lateral views, anterior to left, dorsal to top. Arrowheads indicate green fluorescent protein (GFP) expressing cells. Conserved non‐coding element 6 (CNE6) (A) induced GFP expression in blood precursor cells and CNE11 (C) in developing notochord. CNE6 and CNE11 do not induce reporter gene expression in pectoral fin (B and D). b, blood precursor cells; nc, notochord; y, yolk.

In this study we sought to evaluate the regulatory potential of CNE6 and CNE11 in the pectoral fin of the zebrafish to investigate whether the appendage specific activity of these two enhancers is conserved across bony vertebrates. For this purpose, we used the *Tol2* vector based strategy and amplified CNE6 and CNE11, which are 862 bp and 1185 bp, respectively (Abbasi *et al*. 2010). Interestingly, neither CNE6 nor CNE11 could induce reporter gene expression in the developing pectoral fin of the fish at day 2 or day 3 of development (Fig. 4B,D). Instead, the *Tol2* assay indicated the prominent expression domain for CNE6 to be blood precursor cells, and for CNE11 the notochord (Fig. 4A,C). Therefore, our data reflect that *GLI3*‐associated *cis*‐regulators underwent both genetic and developmental alteration during vertebrate evolution, acquiring prominently divergent roles in tetrapod and bony fish lineages.

### Human‐fish conserved CNE8 is unable to upregulate reporter expression in transgenic zebrafish assays

The human‐fish conserved intronic interval CNE8 was identified in our previously reported comparative analyses of the *GLI3* locus. However, the functionality of this element was not tested previously (Abbasi *et al*. 2007). In this study we sought to investigate the enhancer potential of this human intronic patch of 574 bp sequence located in intron‐10 of the *GLI3* gene. This human interval spans a human‐fish conserved core sequence of 144 bp. Approximately 200 embryos were screened for GFP reporter activity at set time points for both the co‐injection and *Tol2* based transgenic assays. However, both assays indicate that CNE8 is unable to activate reporter gene expression.

### Luciferase reporter assays of *GLI3*‐associated CNEs

Our *in vivo* data demonstrate that both CNE13 and CNE14 can function for tissue‐specific expression of GFP reporters. In order to further examine activities of these CNEs and compare them with activities of other previously‐identified CNEs, we set up *in vitro* luciferase reporter assays. We used the NIH3T3 cell line, a mouse mesenchyme cell line, which can respond to Hedgehog signaling, a major regulator of GLI function (Kim *et al*. 2010).

**Figure 5:**
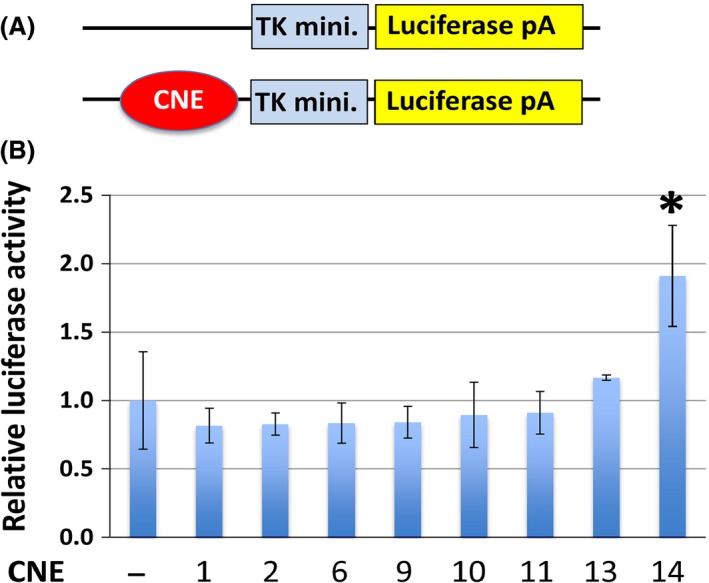
Luciferase reporter assays of *GLI3*‐associated conserved non‐coding elements (CNEs) in the NIH3T3 cell line. (A) Schematic drawings of the luciferase constructs. CNEs were cloned into a pGL3 luciferase vector with the TK minimum promoter (TK mini). (B) Luciferase reporter activities without CNE and with indicated CNEs in the NIH3T3 cell line. Shown is average ± standard deviation. *P < 0.05.

We observed upregulation of reporter activities by CNE14, which is consistent with GFP reporter activation in the fin bud in an *in vivo* assay. Contrary to this, we did not detect changes in reporter activities by CNE13 as well as other CNEs (Fig. 5). These results indicated that CNE13 and CNE14 possess distinct functionalities.

## Discussion

Genomic comparison of a diverse set of vertebrate species revealed many conserved non‐coding elements (CNEs) that appear to have been unchanged throughout vertebrate evolution (Woolfe *et al*. 2005; Pennacchio *et al*. 2006). These elements are associated with genes that coordinate development and have been proposed to act as transcriptional enhancers (Paparidis *et al*. 2007; Pauls *et al*. 2012; Minhas *et al*. 2015). Despite their extreme sequence conservation in vertebrates, the expression pattern driven by these enhancers may vary in different vertebrate lineages (Ariza‐Cosano *et al*. 2012).

Previously, comparative analysis of the human and fugu *GLI3* locus has identified 12 *GLI3*‐associated CNEs distributed throughout the introns of the GLI3 gene (Abbasi *et al*. 2007). When tested *in vitro* in a human cell line that expresses endogenous *GLI3*, these intra‐*GLI3* CNEs had different activities: activators, repressors or no change of luciferase reporter activities. These differential activities provide strong evidence in favor of assigning *GLI3*‐specific regulatory potential to these intronic CNEs. Furthermore, many aspects of reporter gene expression induced in transgenic mouse and zebrafish embryos by the human intronic *GLI3* CNEs represent prominent sites reported for endogenous mouse and zebrafish *Gli3*/*gli3* (Abbasi *et al*. 2007, 2010, 2013; Paparidis *et al*. 2007; Coy *et al*. 2011).

In the present study we identified two novel ancient non‐coding intervals in intron‐3 and 4 of *GLI3* by employing comparative sequence analysis of the *GLI3* containing locus of humans, mice, chickens, lizards, fugu, and zebrafish (Fig. 1 and Fig. S1). The *in vivo* regulatory potential of these two novel CNEs (named CNE13 and CNE14) was tested via a zebrafish co‐injection assay (Woolfe *et al*. 2005) and *Tol2*‐based transgenesis in zebrafish (Fisher *et al*. 2006). CNE13‐controlled GFP expression was strongly expressed in the hindbrain, a finding consistent with the known roles and expression pattern of GLI3 during CNS development (Table 2). Therefore, taking into consideration our previously reported *GLI3* enhancers (CNE1, 2, 6, 9 and 11), we identified six *GLI3*‐intronic enhancer regions in total that control reporter gene expression in the developing neural tissues of mouse and zebrafish embryos (Fig. 1). With multiple independent enhancers controlling early CNS patterning, *Gli*3 resembles other key developmental genes that feature a high level of complexity in their genetic regulatory mechanisms governing CNS patterning (Nobrega *et al*. 2003; Kimura‐Yoshida *et al*. 2004).

**Table 2:** Conserved endogenous expression of Gli3 in vertebrates. The mouse‐zebrafish conserved endogenous expression pattern of Gli3 in various anatomical domains is shown in this table. The “+” sign shows the expression in individual organism specific tissue domains, and the last column provides the source of literature, addressing the Gli3 expression pattern

Tissue	Mouse	Zebrafish	Source
Forebrain	+	+	Huang *et al*. (2008), Tyurina *et al*. (2005)
Midbrain	+	+	Tyurina *et al*. (2005), Aoto *et al*. (2002)
Hindbrain	+	+	Tyurina *et al*. (2005), Aoto *et al*. (2002)
Spinal cord	+	+	Tyurina *et al*. (2005), Coy *et al*. (2011)
Notochord	+	+	Tyurina *et al*. (2005), Mo *et al*. (1997)
Eye	+	+	Tyurina *et al*. (2005), Nakashima *et al*. (2002)
Optic vesicle	+	+	Tyurina *et al*. (2005), Nakashima *et al*. (2002)
Limb/fin	+	+	Büscher *et al*. (1997), Tyurina *et al*. (2005), Muto *et al*. (2014)
Muscle cells	+	+	Tyurina *et al*. (2005), McDermott *et al*. (2005)

Within the somites, Gli3 expression is widespread and known to play a vital role in epaxial and hypaxial myotome formation (McDermott *et al*. 2005). Consistent with such roles, CNE13 upregulates reporter expression predominantly within the muscles of developing zebrafish (Table 2 and Fig. S2). Together with our previously reported *GLI3*‐associated enhancers, it thus appears that at least three independent enhancer regions (CNE9, 11 and 13) govern reported GLI3 expression and function during muscle formation (Fig. 1).

Based on the *Tol2* transgenesis methodology, in the present study we report two novel appendage‐specific enhancers residing within *GLI3* intronic intervals. In addition to the hindbrain and muscles another prominent activity domain of CNE13 in zebrafish was the pectoral fin. CNE13‐induced robust and reproducible reporter expression in the pectoral fin at 48 hpf (Fig. 2E). Similarly, the second novel element (CNE14), which resides within intron‐3 of *GLI3*, induced widespread reporter expression in the pectoral fin bud after 48 hpf (Fig. 3). Interestingly, CNE14‐induced enhancer activity was explicitly restricted to the pectoral fin and a careful examination revealed no significant reporter expression in any of the other developmental compartments of zebrafish embryos at day 2 or day 3 of development. Furthermore, *in silico* analysis of CNE13 and CNE14 predicts human‐fugu conserved binding sites for a number of developmentally important transcription factors that are known to be co‐expressed with Gli3 during embryogenesis (Table 1).

In addition to CNE13 and 14 (present study), we previously defined two independent genomic intervals (named CNE6 and 11) (Fig. 1) regulating reporter expression in distinct domains of developing mice and chicken limbs (Abbasi *et al*. 2010). In the present study, we investigated the using a very reliable *Tol2* transgenesis methodology. In contrast to reporter expression data from mice, neither CNE6 nor CNE11 could induce reporter gene expression in the zebrafish fin (Fig. 4). Given the fact that both of these enhancers are moderately conserved down to teleost fish (Abbasi *et al*. 2007), the sharp difference in their function among mice and fish was surprising. We therefore speculate that CNE6 and CNE11 might have acquired novel appendage‐specific activity during the course of tetrapod evolution through a progressive gain of novel transcriptional factor binding sites around the anciently conserved core sequence (Abbasi *et al*. 2007, 2010). This may have allowed fine‐tuning of gene expression differentially in the tetrapod lineage, congruent with their complex developmental and anatomical needs. Studies have already shown that transformation from fin to limb required alterations in the genetic and developmental tool kit during tetrapod evolution (Shubin *et al*. 2006; Sakamoto *et al*. 2009; Abbasi 2011; Yano & Tamura 2013). Moreover, the experimental variations between zebrafish and mouse, including the use of diverse minimal promoters (*c‐fos* and *β‐globin*), reporter genes (GFP and *lacZ*), transgenesis techniques (*Tol2* transposon and pro‐nuclear), and endogenous characteristics associated to each transgenic system (transparent and opaque) cannot be neglected.

During early embryonic development of the tetrapod limb, GLI3 plays multiple roles: Shh‐independent polarization of nascent limb bud (Welscher *et al*. 2002a; Osterwalder *et al*. 2014) and regulating anteroposterior patterning of the autopod by counteracting Shh signaling (Wang *et al*. 2000; Litingtung *et al*. 2002; Welscher *et al*. 2002b). Gli3 also regulates specification of skeletal precursors for development of specific limb skeletal elements (Barna *et al*. 2005; Robert & Lallemand 2006). These distinct roles of GLI3 during limb development suggest that a complex *cis*‐regulatory landscape might be instrumental in deploying *GLI3* product at different time/domains of limb development. Accordingly, CNE13 and CNE14 exhibited distinct activities in the *in vitro* luciferase reporter assays, supporting the notion of complex and cellular context‐dependent regulation of *GLI3* expression. The exact molecular mechanisms that define the different activities between CNE13 and CNE14 in the developing fin remains to be elucidated. It is conceivable that endogenous factors to drive CNE13 in the fin bud and hindbrain would not be present in NIH3T3 cells, while CNE14 could regulate reporter expression both in NIH3T3 cells and developing fin buds. Similarly, CNE‐1, 2, 6, 9 and 11, which also exhibited activities to drive reporter gene expression in neural tissue in previous studies, were also not activated in NIH3T3‐based luciferase reporter assay. Taken together with our previously reported data, we defined four independent *GLI3*‐intronic intervals (CNE6, 11, 13 and 14) regulating reporter expression in the developing limb/fin bud (Fig. 1) (Abbasi *et al*. 2010).

Here, we propose that the spatial and temporal activity of novel enhancers reported in the present study (CNE13 and CNE14) must also be investigated thoroughly in mice or chicken. Experimental data from such tetrapod model animals would further define the spatiotemporal aspects of CNE13 and CNE14 activity during anterior‐posterior polarity of the limb and patterning of the CNS.

## Conclusions

Taken together with our previous reports, the identification of two novel *cis*‐regions at the *GLI3* locus reflects that *GLI3* harbors multiple *cis*‐acting regulatory modules that participate in an overlapping fashion during development of vertebrate neural tube, limb, and muscles. A subset of these *cis*‐regulators is dual in nature and demonstrated context‐dependent regulation of *GLI3* expression. These findings suggest that even though *GLI3* in tetrapod and teleost shared multiple evolutionarily conserved *cis*‐acting regulators, the target site specificity of some of these elements has diverged significantly between these two lineages. This sort of functional differentiation might have been achieved either through changes in the overall span of enhancers or through the turnover of transcriptional factor binding site inputs. Furthermore, this complex catalogue of *GLI3*‐associated *cis‐*regulators will help in understanding the genetic basis of those potential human birth defects that cannot be attributed to a mutation in coding sequence of *GLI3*. In such cases, these *cis*‐regulatory modules can be investigated among those mutations that can potentially affect the space and time availability of the *GLI3* transcript during embryogenesis.

## Author contributions

A.A.A. and S.S.A. conceived the project. A.A.A., G.E. and Y.K designed the experiments. Computational analysis were performed by S.A., S.S.A., and R.M. The *in vivo* experiments were performed by S.A. and R.M. *In vitro* assays were performed by Y.K and N.L. The manuscript was written by A.A.A., R.M., S.A., and Y.K

## Conflict of interests

The authors declare that they have no competing interests.

## Supporting information


**Fig. S1.** Conservation of the non‐coding elements across the *GLI3* locus.

**Fig. S2.** Schematic representation of GFP expression induced by *GLI3* associated CNE13 in zebrafish embryos at day 2 (~24 hpf) and day 3 (~48 hpf).
