# Supplementary figures and images for: Identification and functional characterization of novel transcriptional enhancers involved in regulating human GLI3 expression during early development
Source: Dev Growth Differ. 2015 Oct 14;57(8):570–80. doi: 10.1111/dgd.12239 (PMC4609622; doi:10.1111/dgd.12239)

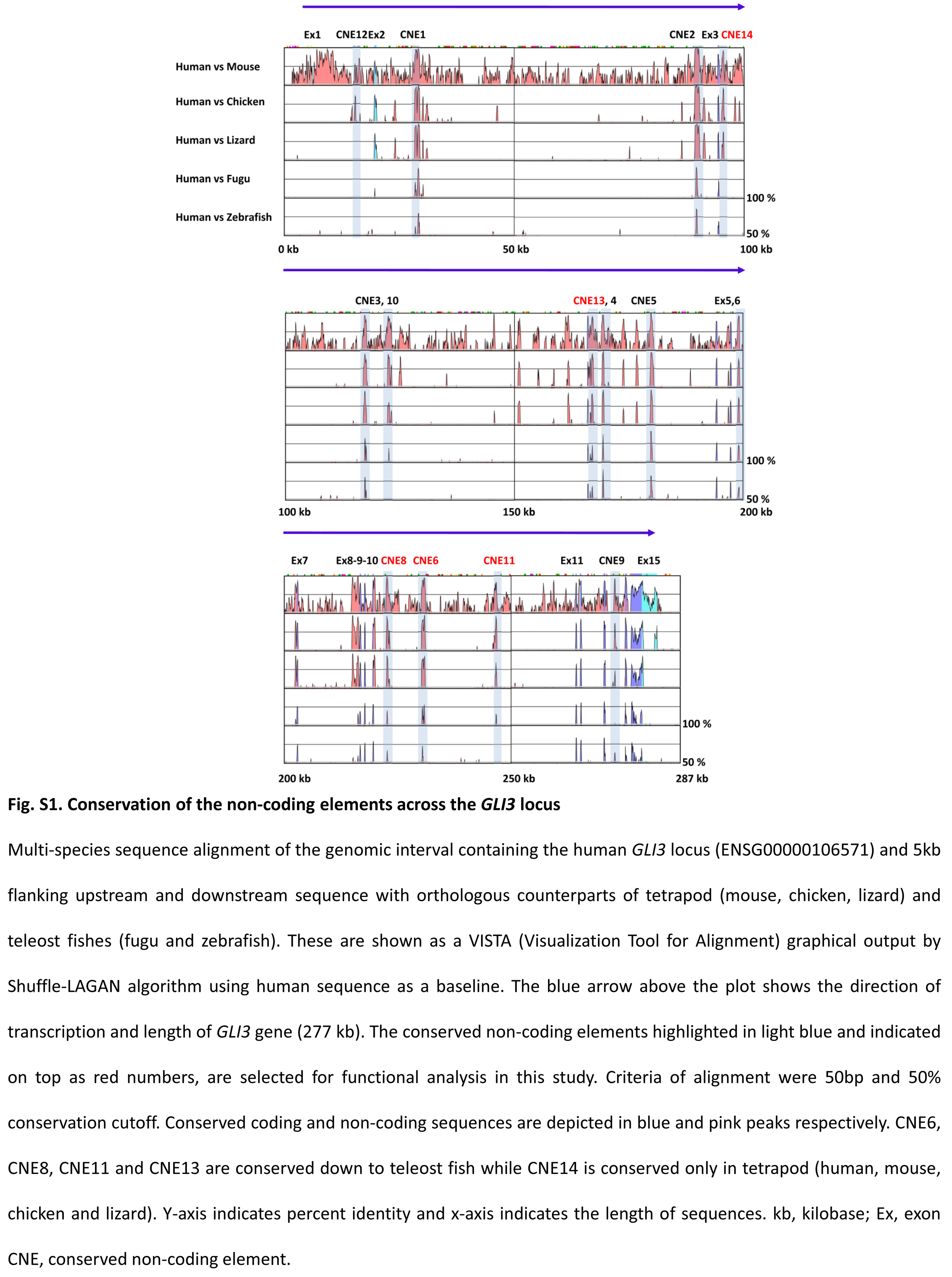

Supplement: Supplementary file 1 — Fig. S1. Conservation of the non‐coding elements across the GLI3 locus. [file DGD-57-570-s001.tif]

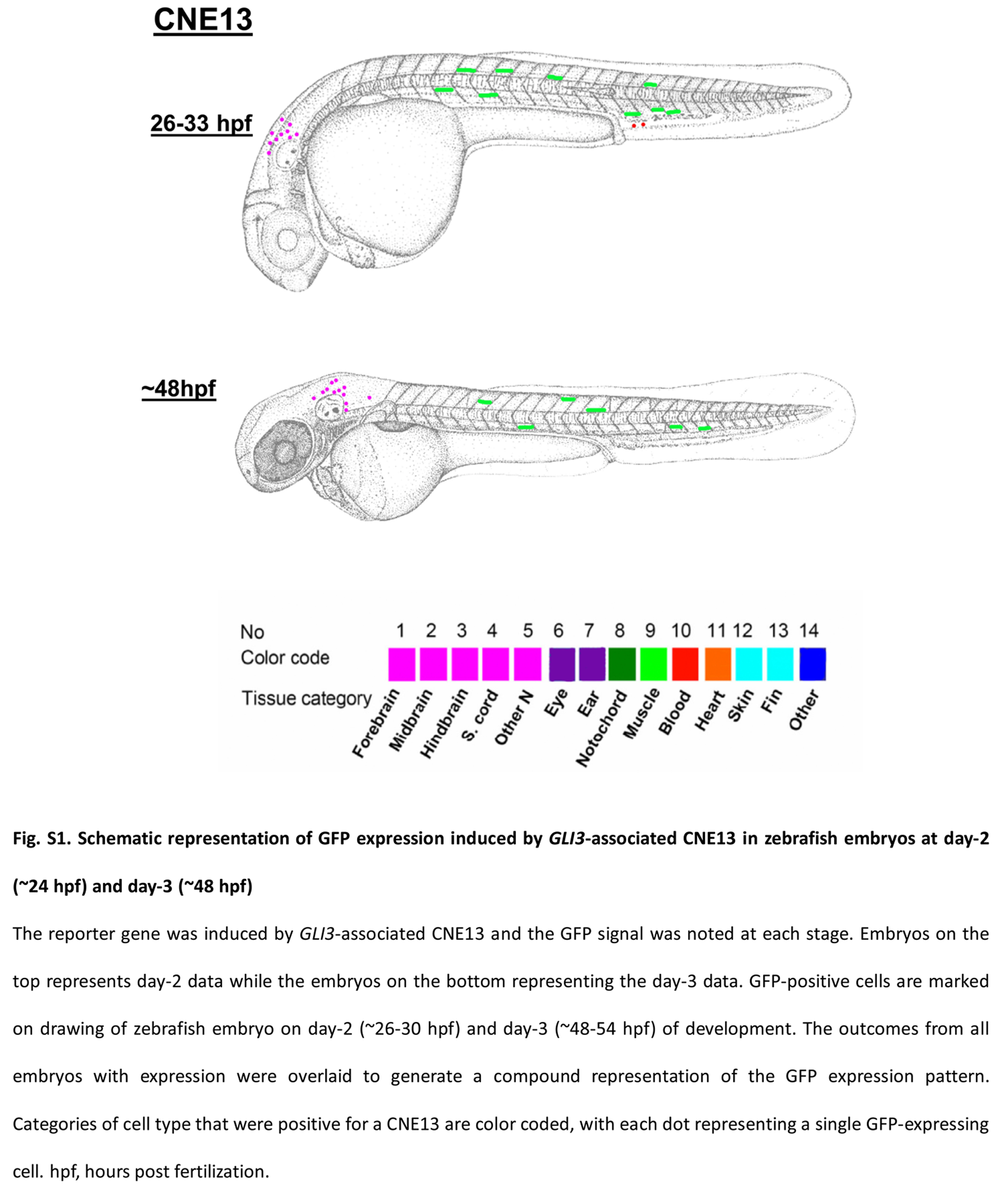

Supplement: Supplementary file 2 — Fig. S2. Schematic representation of GFP expression induced by GLI3 associated CNE13 in zebrafish embryos at day 2 (~24 hpf) and day 3 (~48 hpf). [file DGD-57-570-s002.tif]
